# Implant Surface Characteristics and Peri-Implant Outcomes: A Systematic Review of Clinical and Microbiological Evidence

**DOI:** 10.3390/bioengineering13030299

**Published:** 2026-03-03

**Authors:** Gianna Dipalma, Grazia Marinelli, Paola Bassi, Rosalba Lagioia, Antonio Rizzo, Sara Savastano, Francesco Inchingolo, Cristina Grippaudo, Angelo Michele Inchingolo, Alessio Danilo Inchingolo

**Affiliations:** 1 Department of Interdisciplinary Medicine, University of Bari “Aldo Moro”, 70121 Bari, Italy or gianna.dipalma@unimi.it (G.D.); graziamarinelli@live.it (G.M.); paola.bassi@uniba.it (P.B.); rosalba.lagioia@uniba.it (R.L.); antonio.rizzo@uniba.it (A.R.); sara.savastano@uniba.it (S.S.); or angelomichele.inchingolo@unimi.it (A.M.I.); alessiodanilo.inchingolo@uniba.it (A.D.I.); 2 Department of Biomedical, Surgical and Dental Sciences, Milan University, 20122 Milan, Italy; 3 Dipartimento Universitario Testa Collo ed Organi di Senso, Università Cattolica del Sacro Cuore, 00168 Rome, Italy; 4 UOC Clinica Odontoiatrica, Dipartimento di Neuroscienze, Organi di Senso e Torace, Fondazione Policlinico Universitario A. Gemelli IRCCS, 00168 Rome, Italy

**Keywords:** dental implants, implants surface modification, peri-implantitis, marginal bone loss, implant surface roughness, peri-implant outcomes, osseointegration, antibacterial coatings

## Abstract

**Background:** Implant surface characteristics have been extensively investigated for their potential influence on osseointegration and peri-implant tissue stability. However, their actual clinical relevance in the prevention and progression of peri-implant diseases remains controversial. This systematic review aimed to synthesize the available clinical and microbiological evidence on the impact of different implant surface characteristics and surface modifications on peri-implant outcomes. **Materials and Methods:** Conducted according to PRISMA and registered in PROSPERO, an electronic search of PubMed, Scopus, and Web of Science (2015–2025) identified clinical studies assessing associations between implant surface characteristics/modifications and peri-implant clinical, radiographic, microbiological, or biomolecular outcomes. Risk of bias was evaluated using ROBINS-I. **Results:** Thirteen studies (randomized, controlled, and cohort designs) were included. Most trials reported minimal differences in marginal bone loss and peri-implant parameters across surfaces. Potential advantages were mainly observed during early healing or in compromised bone. Long-term evidence emphasized the predominance of patient- and site-related risk factors. Microbiological outcomes were scarce and heterogeneous. **Conclusions:** Implant surface modifications appear to exert a limited and context-dependent influence on peri-implant outcomes. Long-term peri-implant health is primarily driven by multifactorial interactions involving host, microbial, and clinical factors rather than surface characteristics alone.

## 1. Introduction

Dental implants are a predictable and widely adopted treatment option for the rehabilitation of partially or fully edentulous patients, with high long-term survival rates reported across different clinical scenarios [1,2,3]. Nevertheless, peri-implant diseases, ranging from peri-implant health to peri-implant mucositis and peri-implantitis, remain a major clinical challenge [4,5,6]. Peri-implantitis, in particular, is a biofilm-associated inflammatory condition characterized by progressive peri-implant bone loss and represents one of the main causes of late implant failure [7,8,9,10].

The pathogenesis of peri-implant diseases is multifactorial and involves the complex interaction between microbial biofilms, host immune–inflammatory response, and local as well as systemic risk factors [11,12,13]. In this context, implant surface characteristics have been extensively investigated because of their potential influence on both osseointegration and microbial colonization [14,15,16,17]. Surface topography, chemistry, energy, and wettability are known to modulate protein adsorption, cell adhesion, osteoblastic differentiation, and bacterial attachment, thereby potentially affecting marginal bone stability and peri-implant tissue health [18,19,20].

From a biological perspective, moderately rough surfaces have been shown to enhance bone-to-implant contact (BIC) and early mechanical stability compared with minimally rough or machined surfaces [21,22,23]. This has led to the widespread adoption of sandblasted and acid-etched (SLA) surfaces and their subsequent modifications, including hydrophilic treatments, anodization, laser micro-grooving, and bioactive or ionic coatings [24,25,26]. More recently, nano-scale modifications and chemically activated surfaces have been introduced with the aim of accelerating the transition from primary to secondary stability, especially in compromised bone conditions, and supporting early or immediate loading protocols [27,28,29,30].

However, while surface roughness and chemical activation may promote faster and stronger osseointegration, concerns have been raised regarding their potential role in increasing susceptibility to peri-implant inflammation [31,32,33]. Experimental and clinical data suggest that rougher surfaces may favor bacterial retention once exposed to the oral environment, potentially exacerbating peri-implant tissue breakdown under conditions of inadequate plaque control [34,35,36,37]. Long-term observational studies in patients with a history of periodontitis have reported a higher incidence of peri-implantitis around moderately rough surfaces compared with minimally rough implants, highlighting the importance of patient-related risk factors and maintenance protocols [38,39,40].

For example, in a 5-year split-mouth randomized clinical trial in periodontitis-susceptible patients, peri-implantitis (PPD ≥ 5 mm + BoP + radiographic bone loss ≥ 2.5 mm) was diagnosed in 12/42 moderately rough TiUnite implants versus 3/41 minimally rough turned implants (*p* < 0.01), while cumulative survival was 100% and 97.6%, respectively [41]. Consistently, a pooled retrospective cohort analysis (630 implants; mean follow-up 13 years) found that rough implant surfaces (Sa > 1 µm) were associated with higher odds of peri-implantitis compared with machined surfaces (OR 4.877, 95% CI 1.701–13.980), after accounting for confounders such as smoking and implant location [42].

Clinical trials comparing different surface treatments frequently report minimal or no statistically significant differences in marginal bone loss during short- and medium-term follow-up [43,44,45,46]. Randomized and split-mouth studies evaluating conventional SLA surfaces versus hydrophilic or chemically modified surfaces generally show comparable radiographic outcomes at 1 to 3 years, with marginal bone changes remaining within clinically acceptable limits [47,48,49]. Similarly, modifications limited to the transmucosal or collar region, such as anodization or laser micro-grooving, do not consistently translate into measurable improvements in peri-implant bone stability or soft tissue parameters [50,51,52].

Conversely, specific surface modifications have shown promising results in selected clinical contexts [53,54,55]. Bioactive or nano-superhydrophilic surfaces appear to stabilize implant stability quotient (ISQ) values during the critical early healing phase, particularly in low-density bone, potentially supporting earlier functional loading without adverse effects on marginal bone levels [56,57,58,59]. Ionic modifications, such as calcium-incorporated surfaces, have been associated with reduced early marginal bone loss in challenging scenarios like transalveolar sinus augmentation [60,61,62]. In addition, antibacterial strategies targeting the internal components of the implant–abutment complex have demonstrated a reduction in bacterial load at early follow-up, suggesting a potential adjunctive role in controlling peri-implant microbial contamination [63,64,65].

In parallel, antimicrobial coatings are increasingly investigated as adjunctive approaches to limit early bacterial adhesion and biofilm development on implant- and abutment-related surfaces. Recent overviews on polymeric dental nanomaterials with antimicrobial activity report that nanoparticle-containing polymer composites (including silver-, chitosan-, and titanium oxide–based systems) have been explored across several dental fields, including dental implantology and dental prosthetics, with the aim of improving resistance to microbial colonization [66].

Among polymeric candidates, chitosan-based coatings are of particular interest due to their biocompatibility and chemical versatility. In this context, L-arginine functionalization of chitosan derivatives has been proposed to enhance antimicrobial performance for potential use as coatings on implantable devices; notably, arginine-modified chitosan derivatives demonstrated the highest antibiofilm activity across multiple microbial strains, reaching complete or near-complete inhibition at the highest tested concentrations [67]. Although these approaches are largely supported by preclinical evidence, they provide a rationale for considering antimicrobial coatings within the broader landscape of surface engineering strategies aimed at balancing osseointegration with infection control.

Broadly, coating approaches for titanium dental implants can be grouped into polymeric matrices and organic–inorganic (including hybrid) matrices, each with distinct advantages and limitations. Polymeric coatings (e.g., chitosan- or hydrogel-based layers, multilayers, and functional polymers) are attractive because they can be processed under mild conditions and offer high chemical tunability, enabling the incorporation/immobilization of antimicrobial agents and the design of controlled local release systems [68,69]. Their main drawbacks are related to long-term stability in the oral environment, including degradation or swelling, potential changes after sterilization, and the risk of limited adhesion or wear-related loss over time [70]. In contrast, inorganic or organic–inorganic matrices (e.g., calcium phosphate/HA, oxide-based, sol–gel derived, and hybrid coatings) can provide higher hardness and chemical stability and may improve osteoconductivity and corrosion protection; however, brittleness, cracking, interfacial stresses, and delamination remain relevant concerns for some ceramic coatings, potentially affecting long-term reliability. Hybrid organic–inorganic strategies aim to combine the mechanical robustness of the inorganic phase with the functional versatility of the organic component, but manufacturing complexity and translation-to-market challenges still represent key barriers [71,72].

Despite these findings, the overall clinical relevance of implant surface characteristics in the prevention and progression of peri-implant diseases remains controversial [73,74,75]. Large retrospective cohorts and comparative clinical studies indicate that patient-related factors, such as smoking, history of periodontitis, plaque accumulation, and site-specific conditions, often exert a stronger influence on peri-implantitis development than surface topography alone [76,77,78]. Moreover, microbiological and biomolecular outcomes, including inflammatory mediator levels in peri-implant crevicular fluid, have been investigated in a limited number of studies, providing preliminary but inconclusive evidence regarding surface-dependent inflammatory responses [79,80,81].

Taken together, the available evidence suggests that implant surface modifications may influence specific biological and clinical outcomes, particularly during early healing or under compromised conditions [82,83,84]. However, peri-implant diseases appear to be driven by a complex and multifactorial interplay between implant characteristics, microbial challenge, and host susceptibility [85,86,87,88]. Heterogeneity in study designs, surface classifications, disease definitions, follow-up durations, and outcome measures further complicates the interpretation of existing data [89,90,91].

Therefore, a systematic synthesis of the clinical and microbiological evidence focusing on implant surface characteristics and peri-implant outcomes is warranted [92,93,94]. The present systematic review aims to evaluate and critically appraise the available comparative clinical studies investigating the relationship between different implant surface modifications and peri-implant outcomes, including marginal bone loss, peri-implant health parameters, and disease occurrence, with the objective of clarifying the actual clinical impact of surface characteristics within a multifactorial risk framework [95,96,97].

## 2. Materials and Methods

### 2.1. Protocol and Registration

This review was conducted in accordance with PRISMA (Preferred Reporting Items for Systematic Reviews and Meta-Analyses) guidelines, and it was filed with the number 1301140 on PROSPERO (The International Prospective Register of Systematic Reviews). The review was designed to evaluate the comparative clinical evidence on the impact of dental implant surface characteristics and surface modifications (including differences in topography, chemistry, wettability, bioactive coatings, ionic modifications, and antibacterial strategies) on peri-implant outcomes, encompassing clinical and radiographic parameters (e.g., bleeding on probing, probing depth, marginal bone loss), as well as microbiological and biomolecular findings when available.

### 2.2. Search Processing

We focused our search on English-language publications published between 1 January 2015 and 1 December 2025 in PubMed, Scopus, and Web of Science that were relevant to our topic. In the search, the Boolean keywords (dental implant) AND (surface OR coating OR antibacterial OR antimicrobial) AND (peri-implantitis OR periimplantitis OR marginal bone loss OR biofilm) were utilized. We picked these terms because they best characterized the purpose of our study, which was to learn more about dental implants, surface modifications, and peri-implant outcomes (Table 1).

### 2.3. Inclusion Criteria

Three reviewers reviewed all relevant publications using the following criteria: (1) only human subjects research, (2) full text, and (3) scientific studies evaluating the relationship between dental implant surface characteristics or surface modifications and peri-implant clinical, radiographic, microbiological, or biomolecular outcomes.

The PICOS model was created using the following steps:

1. Criteria: application in the present study;
2. Population: human subjects rehabilitated with dental implants;
3. Intervention: evaluation of different implant surface characteristics or surface modifications;
4. Comparison: comparison between implants with different surface characteristics or conventional/reference surfaces;
5. Outcome: evaluation of peri-implant outcomes, including marginal bone loss, peri-implant health parameters, peri-implant mucositis/peri-implantitis occurrence, and, when available, microbiological or biomolecular findings;
6. Study design: randomized controlled trials (RCT), controlled clinical trials, prospective observational studies, retrospective cohort studies, and comparative clinical studies.

### 2.4. Exclusion Criteria

Exclusion criteria included: (i) animal studies; (ii) in vitro or ex vivo investigations; (iii) systematic reviews and meta-analyses; (iv) studies not aligned with the objectives of the review, including those not providing comparative evaluation of implant surface characteristics or not reporting relevant peri-implant clinical and/or radiographic outcomes; and (v) studies with insufficient sample size or inadequate methodological design to support meaningful clinical interpretation.

For the purpose of this review, an insufficient sample size was defined as studies enrolling fewer than 10 patients overall, fewer than 20 implants in total, or, in comparative designs, fewer than 10 patients per study arm. Additionally, studies evaluating disease-related outcomes (e.g., peri-implantitis incidence) without reporting any sample size justification or power consideration were carefully assessed and excluded when the limited cohort size prevented meaningful clinical inference. Methodological inadequacies leading to exclusion included: (a) non-comparative designs (e.g., single-arm case series) or absence of a control/comparator group; (b) unclear or non-reproducible definition and/or measurement of peri-implant outcomes (e.g., marginal bone loss measurement protocol not described or peri-implantitis case definition not provided); (c) follow-up limited to the immediate postoperative period or too short to evaluate the reported outcomes (e.g., no clinical/radiographic assessment beyond baseline or <3 months after placement/loading); (d) insufficient reporting to allow data extraction or appraisal (e.g., missing sample size/implant numbers per group). These thresholds were established to exclude exploratory or anecdotal investigations while retaining clinically relevant comparative trials and cohort studies.

### 2.5. Data Processing

Author conflicts regarding article selection were addressed and resolved through discussion and consensus among the reviewers.

### 2.6. Article Identification Procedure

Appropriateness was evaluated independently by two reviewers, P.B. and R.L. To make more papers available for full-text analysis, a second manual search was carried out. After evaluating English-language articles that satisfied the inclusion requirements, duplicates and non-qualifying items were noted and their exclusions explained.

### 2.7. Study Evaluation

The reviewers separately evaluated the article data using a specific electronic form established according to the following categories: authors, year of study, aim of the study, materials and methods, and results.

### 2.8. Quality Assessment

The quality of the included papers was evaluated by two reviewers, P.B. and R.L., using the ROBINS-I approach. In non-randomized studies evaluating the health effects of two or more medications, ROBINS-I was created to assess the risk of bias. A bias degree was assigned to each of the seven criteria that were examined. In the event of a disagreement, the third reviewer, F.I., was consulted until a resolution was reached. Any disagreements or disputes amongst reviewers were settled through dialog and consensus-building in order to improve the evaluations’ objectivity and consistency. A third reviewer made the final decision in cases where there was no agreement. By identifying the advantages and disadvantages of the evidence base, it helped to provide a more accurate evaluation of the results’ quality and dependability. By accounting for the likelihood of bias, the authors of this review were able to draw more informed interpretations and conclusions based on the evidence presented.

## 3. Results

**Figure 1:**
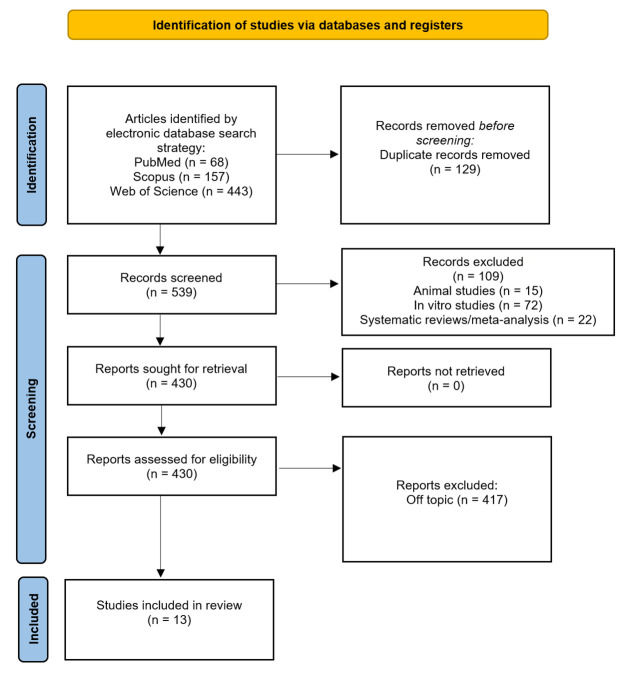
PRISMA flowchart of the literature search and article inclusion process.

The database search identified a total of 668 records (PubMed n = 68; Scopus n = 157; Web of Science n = 443). After removal of 129 duplicates, 539 records were screened. During screening, 15 animal studies, 72 in vitro/ex vivo investigations, and 22 systematic reviews or meta-analyses were excluded. An additional 417 articles were excluded because they were not aligned with the objectives of the present review, primarily due to insufficient sample size, non-comparative designs or absence of a control/comparator group, unclear outcome definitions/measurements, lack of comparative assessment of implant surface characteristics according to the predefined thresholds described in the exclusion criteria, or absence of relevant peri-implant clinical/radiographic outcomes. Ultimately, 13 studies were included in the qualitative synthesis (Figure 1). The results of each investigation were shown in Table 2.

### Characteristics of Included Studies

The 13 studies analyzed in this review investigated the influence of implant surface characteristics or surface modifications on peri-implant clinical, radiographic, microbiological, and biomolecular outcomes, using a variety of clinical study designs.

Sample sizes varied widely across the included studies, ranging from 18 to 600 participants and from 35 to 630 implants (Table 2). Most randomized or controlled trials enrolled relatively small cohorts and were primarily powered for early or surrogate outcomes (e.g., marginal bone loss, peri-implant soft-tissue indices, or implant stability measures) rather than for peri-implantitis incidence, which was more often addressed in long-term retrospective or pooled cohort investigations.

Importantly, several included trials had short follow-up (e.g., 6–12 months) and mainly reported early radiographic changes (MBL) and/or stability measures; therefore, these outcomes should be interpreted as early peri-implant tissue responses rather than definitive peri-implantitis endpoints.

Six studies were randomized clinical trials, including split-mouth or controlled designs, evaluating differences in marginal bone loss, implant stability, and peri-implant soft tissue parameters between distinct surface types. In particular, Canullo et al. (2024) assessed nano-superhydrophilic bioactive surfaces in poor-quality bone, focusing on the transition from primary to secondary stability [98]. Guarnieri et al. (2019) evaluated submerged versus nonsubmerged laser-microgrooved implants in posterior areas, while Vílchez et al. (2025) compared hydrophilic modified SLA surfaces with conventional SLA implants [99,100]. Additional randomized trials investigated calcium-phosphate–coated versus uncoated SLA implants (Ko et al., 2019), anodized versus machined implant collars (Longhi et al., 2025), and different loading protocols using thermo-chemically treated implant surfaces (Albertini et al., 2021) [101,102,103]. Follow-up durations in randomized studies ranged from 6 months to 5 years. Short-term follow-up studies mainly captured early marginal bone remodeling and peri-implant soft-tissue responses rather than late peri-implantitis endpoints.

Three studies were prospective or controlled clinical investigations focusing on specific surface-related strategies and early biological responses. Carinci et al. (2019) examined the effect of an antibacterial internal implant coating on peri-implant bacterial load using microbiological analysis, while other controlled clinical studies evaluated peri-implant bone and soft tissue outcomes associated with different surface or component modifications (Anitua et al., 2017; Şener-Yamaner et al., 2017) [104,107,108].

Four studies adopted retrospective or pooled cohort designs, providing long-term data on peri-implantitis occurrence and associated risk factors. Raes et al. (2018) conducted a 5-year split-mouth randomized clinical trial in patients with a history of severe periodontitis, comparing minimally and moderately rough implant surfaces and reporting a higher incidence of peri-implantitis on moderately rough implants [41]. Ferrantino et al. (2022) performed a pooled retrospective cohort analysis showing a significant association between surface roughness, smoking habits, implant site location, and peri-implantitis occurrence [42]. Similarly, Hussain et al. (2024) reported that patient-related factors exerted a stronger influence on peri-implantitis development than surface topography when comparing two major implant systems [105]. A prospective clinical investigation by Gnanajothi et al. (2024) further explored peri-implant outcomes associated with different surface characteristics, reporting limited differences in short-term clinical parameters [106].

Radiographic outcomes, particularly marginal bone loss, represented the most frequently assessed endpoints across the included studies. Clinical parameters such as probing depth, bleeding on probing, plaque indices, and peri-implant disease incidence were evaluated in most investigations, whereas microbiological or biomolecular outcomes were reported in a limited number of studies, mainly focusing on bacterial load and inflammatory markers (Carinci et al., 2019; Raes et al., 2018) [41,104].

Overall, the included studies demonstrated substantial heterogeneity in study design, implant systems, surface classifications, follow-up duration, and outcome definitions, precluding quantitative meta-analysis. Nevertheless, together they provide clinically relevant comparative evidence regarding the role of implant surface characteristics within a multifactorial peri-implant disease framework, in which surface-related effects interact with patient- and site-related risk factors (Table 3).

## 4. Discussion

### 4.1. Implant Surface Characteristics and Clinical–Radiographic Outcomes

The present systematic review synthesized the available comparative clinical evidence on the role of implant surface characteristics and surface modifications in influencing peri-implant clinical and radiographic outcomes [109,110,111]. Overall, the findings from randomized and controlled clinical trials indicate that differences in marginal bone loss between surface types are generally limited, particularly during short- and medium-term follow-up [112,113,114]. Studies comparing conventional moderately rough SLA surfaces with hydrophilic or chemically modified variants consistently reported comparable marginal bone levels at 1 year (Ko et al., 2019; Vílchez et al., 2025) and up to 3 years (Guarnieri et al., 2019) [99,100,101,115,116,117]. Similarly, modifications confined to the transmucosal or collar region, such as anodized or laser-microgrooved collars, did not demonstrate consistent advantages in preserving peri-implant bone or improving soft tissue parameters (Longhi et al., 2025) [102,118,119,120].

These findings suggest that, under controlled clinical conditions, contemporary implant surfaces, regardless of specific chemical or topographical refinements, are capable of achieving predictable osseointegration and marginal bone stability [121,122,123,124,125,126,127,128]. While statistically significant differences were occasionally reported, their magnitude was often small and remained within clinically acceptable thresholds [129,130,131]. This supports the concept that, once a stable bone–implant interface is established, surface-related effects on long-term bone preservation may be overshadowed by other biological and mechanical factors [132,133,134].

Nevertheless, several studies highlighted that surface characteristics may play a more relevant role during early healing or in challenging clinical scenarios [135,136,137]. Nano-superhydrophilic bioactive surfaces demonstrated favorable early stability patterns in low-density bone, facilitating a smoother transition from primary to secondary stability (Canullo et al., 2024) [98,138,139,140]. Likewise, thermo-chemically treated surfaces used under immediate or early loading protocols showed satisfactory clinical and radiographic outcomes without compromising marginal bone levels (Albertini et al., 2021) [103,141,142,143]. These observations indicate that surface engineering may provide meaningful benefits when osseointegration dynamics are critical, even though such advantages do not necessarily translate into superior long-term radiographic outcomes [144,145,146,147].

### 4.2. Implant Surface Roughness, Patient-Related Factors, and Peri-Implant Disease

In contrast to the relatively homogeneous findings reported in short-term randomized trials, studies with longer follow-up and retrospective designs emphasized the multifactorial nature of peri-implant diseases [148,149,150]. A split-mouth randomized clinical trial conducted in patients with a history of severe periodontitis reported a higher incidence of peri-implantitis around moderately rough implant surfaces compared with minimally rough implants (Raes et al., 2018) [41,151,152,153,154]. Specifically, Raes et al. reported that after 5 years, peri-implantitis was diagnosed in 12/42 TiUnite implants compared with 3/41 turned implants (*p* < 0.01), with cumula f 100% (TiUnite) and 97.6% (turned) [41]. This finding has often been cited as evidence of a potential association between increased surface roughness and susceptibility to peri-implant inflammation once the implant surface becomes exposed to the oral environment [155,156,157]. Short-term studies primarily capture early remodeling and soft tissue responses, whereas peri-implantitis is typically a late complication requiring longer observation and standardized case definitions.

However, pooled and retrospective cohort studies provide a more nuanced interpretation [158,159,160]. Large-scale analyses demonstrated that patient-related and site-specific factors, such as smoking habits, previous periodontal disease, implant site location, and maintenance compliance, exert a stronger influence on peri-implantitis development than implant surface characteristics alone [42,105,161,162,163]. In the pooled cohort by Ferrantino et al., rough surfaces were associated with increased peri-implantitis odds (OR 4.877, 95% CI 1.701–13.980), supporting the concept that roughness may modulate disease susceptibility particularly when combined with established patient- and site-related risk factors [42]. Similar conclusions were reported in controlled retrospective investigations, in which peri-implant bone stability and disease occurrence appeared primarily driven by baseline risk profiles rather than by surface topography (Anitua et al., 2017; Şener-Yamaner et al., 2017) [107,108,164,165,166]. A prospective clinical study further supported these findings, reporting only minor short-term differences in peri-implant outcomes between surface types when confounding variables were adequately controlled (Gnanajothi et al., 2024) [106,167,168,169].

Taken together, these data suggest that while implant surface roughness may modulate bacterial adhesion and tissue response under certain conditions, surface characteristics alone are insufficient to explain the onset and progression of peri-implant diseases [170,171,172]. Instead, peri-implantitis should be viewed as the result of a complex interaction between implant-related factors, microbial challenge, host susceptibility, and long-term maintenance [173,174,175,176,177,178].

### 4.3. Microbiological Evidence, Methodological Limitations, and Clinical Implications

Only a limited subset of the included studies investigated microbiological or biomolecular outcomes, underscoring a significant gap in the current evidence base [179,180,181]. An antibacterial strategy targeting the internal components of the implant–abutment complex was associated with a reduction in early bacterial load (Carinci et al., 2019), suggesting that surface or component modifications may have an adjunctive role in controlling peri-implant microbial contamination [104,182,183,184]. However, the heterogeneity of microbiological methods, the lack of standardized outcome measures, and the short duration of follow-up prevent firm conclusions regarding the long-term clinical relevance of these findings [183,185,186].

The interpretation of the available evidence is further complicated by substantial heterogeneity across studies in terms of implant systems, surface classifications, peri-implant disease definitions, and outcome reporting [187,188,189]. Risk-of-bias assessment revealed moderate to serious risk in several non-randomized and retrospective studies, primarily due to confounding and selection bias [190,191,192]. These methodological limitations highlight the need for caution when extrapolating surface-specific effects to routine clinical practice [193,194,195,196,197,198].

In addition, several study-level limitations should be acknowledged. First, many randomized or controlled studies included small samples and/or short follow-up (often 6–12 months), limiting statistical power and preventing robust inference on late endpoints such as peri-implantitis development. Second, outcome reporting was frequently centered on surrogate parameters (e.g., early marginal bone remodeling, ISQ, plaque/bleeding indices), with limited standardization of case definitions for peri-implantitis and heterogeneous radiographic and clinical assessment protocols across studies. Third, blinding was often not feasible and, in non-randomized/retrospective designs, incomplete adjustment for key confounders (e.g., history of periodontitis, smoking, maintenance compliance, prosthetic design) may have influenced the observed associations between surface features and clinical outcomes. Finally, some analyses considered implants as independent units without fully addressing clustering at the patient level, which may bias variance estimates in multi-implant patients.

From a clinical standpoint, the findings of this review suggest that implant surface modifications alone are unlikely to prevent peri-implant diseases or compensate for unfavorable patient-related risk factors [199,200,201,202,203]. While surface engineering continues to evolve and may offer advantages in selected situations, such as compromised bone quality or early loading protocols, long-term peri-implant health appears to depend predominantly on comprehensive risk assessment, meticulous surgical and prosthetic planning, and effective supportive care programs [204,205,206]. Future research should prioritize well-designed randomized clinical trials with standardized peri-implant disease definitions, extended follow-up periods, and integrated clinical, radiographic, and microbiological outcomes to better clarify the true clinical impact of implant surface characteristics within a multifactorial risk framework [207,208,209].

To strengthen the evidence base, future randomized controlled trials should adhere to CONSORT principles and prospectively register protocols, with sample size calculations based on clinically meaningful differences in marginal bone loss and, when peri-implantitis incidence is considered, sufficiently long follow-up to capture late disease onset (ideally at least 3–5 years). Randomization should be stratified for key risk factors (e.g., history of periodontitis, smoking, and maintenance compliance), and analyses should account for clustering when multiple implants are placed in the same patient. Standardized outcome definitions and measurement protocols are essential, including the use of consensus case definitions for peri-implant diseases and core outcome sets for implant dentistry trials (e.g., ID-COSM) [27]. Blinded outcome assessment, calibrated examiners, and transparent reporting of surface characterization and supportive care regimens would further improve comparability and clinical interpretability across studies.

## 5. Conclusions

Based on the available comparative clinical evidence, implant surface characteristics and surface modifications appear to exert a limited and context-dependent influence on peri-implant outcomes. Across randomized and controlled clinical studies, modern implant surfaces (moderately rough SLA surfaces with hydrophilic or chemically modified variants, anodized or laser-microgrooved collars, nano-superhydrophilic bioactive surfaces, thermo-chemically treated surfaces), regardless of specific chemical or topographical modifications, generally demonstrate comparable marginal bone stability and satisfactory peri-implant tissue health under controlled clinical conditions. Surface-related advantages seem to be most evident during early healing phases or in compromised clinical scenarios, such as low-density bone or early loading protocols, whereas their impact on long-term peri-implant disease prevention remains uncertain.

Long-term and retrospective evidence highlights the multifactorial nature of peri-implant diseases, in which patient-related and site-specific factors, including smoking habits, history of periodontitis, oral hygiene, and maintenance compliance, often outweigh the role of implant surface characteristics alone. Microbiological and biomolecular data remain scarce and heterogeneous, preventing definitive conclusions regarding sustained surface-dependent effects on peri-implant inflammation or microbial colonization.

Overall, the findings of this systematic review suggest that implant surface modifications should be considered as adjunctive rather than determinant factors in peri-implant health. Optimal long-term outcomes are more likely to be achieved through comprehensive patient risk assessment, appropriate surgical and prosthetic planning, and effective supportive care programs. Future well-designed randomized clinical trials with standardized peri-implant disease definitions, longer follow-up periods, and integrated clinical, radiographic, and microbiological outcomes are needed to further clarify the true clinical relevance of implant surface characteristics within a multifactorial risk framework.

## Figures and Tables

**Table 1:** Indicators for database searches.

Articles screening strategy	KEYWORDS: (A): dental implant; (B): surface OR coating OR antibacterial OR antimicrobial; (C): peri-implantitis OR periimplantitis OR marginal bone loss OR biofilm.
	Boolean Indicators: (A) AND (B) AND (C).
	Timespan: 1 January 2015, to 1 December 2025.
	Electronic databases: Pubmed; Scopus; Web of Science.

**Table 2:** Characteristics of included studies.

Authors	Year of Study	Type of Study	Aim of the Study	Materials and Methods	Results
Luigi Canullo et al. [98]	2024	Controlled Clinical Trial	Evaluate transition from primary to secondary stability of nano-superhydrophilic bioactive surfaces in poor bone	36 patients; 60 implants in D3–D4 bone; bioactive nano-superhydrophilic vs. conventional moderately rough surface; ISQ measured at placement, 30 and 45 days; MBL evaluated at 6 months after loading.	Bioactive surface showed more stable ISQ during early healing; control group showed transient ISQ reduction. No negative effect on MBL
Renzo Guarnieri et al. [99]	2019	Randomized Clinical Trial	Compare crestal bone loss and soft tissue outcomes of submerged vs. non-submerged laser-microgrooved implants	20 patients; 40 implants; split-mouth design; radiographic CBL and clinical parameters (PD, BOP, plaque index, recession) evaluated up to 3 years after loading.	No significant differences in CBL or peri-implant soft tissue parameters at 3 years between submerged and non-submerged implants.
Blanca Vílchez et al. [100]	2025	Randomized Clinical Trial	Compare MBL between modified hydrophilic SLA and conventional SLA surfaces	122 implants; split-mouth design. Primary outcome MBL at 12 month after loading. Secondary outcomes: PD, BOP, keratinized mucosa width, ISQ.	No clinically relevant differences in MBL or peri-implant soft tissue parameters between surfaces at 12 months.
Kyung-A Ko et al. [101]	2019	Randomized Controlled Trial	Compare CaP-coated vs. uncoated SLA implants	34 patients; 50 implants; randomized double-blind design. Clinical and radiographic evaluation at placement, 3 months and 12 month. Primary outcome: MBL at 1 year	No implant failures. No clinically significant differences in marginal bone level between CaP-coated and conventional SLA implants at 1 year.
Beatrice Longhi et al. [102]	2025	Randomized Clinical Trial	Assess influence of anodized collar vs. machined collar on MBL and peri-implant parameters	30 patients; two adjacent short implants (test and control), Radiographic MBL and clinical parameters (PD, BOP, plaque index) assessed at baseline, 3, 6 and 12 months	No significant differences in MBL or peri-implant indices between anodized and machined collar implants at 12 months.
Matteo Albertini et al. [103]	2021	Randomized Clinical Trial	Compare immediate vs. early loading of thermo-chemically treated implants	21 patients; 35 implants; Immediate loading (1 week) vs. early loading (4 weeks). Radiographic, MBL, ISQ and clinical parameters at 12 months.	No implant loss. No differences in MBL or implant survival between loading protocols
Francesco Carinci et al. [104]	2019	Clinical Trial	Evaluate antibacterial internal implant coating	60 implants; microbiological assessment by Real-Time PCR at 6 months; comparison between coated and non-coated implants.	Significant reduction in bacterial load in coated implants compared to controls at 6 months. Clinical peri-implantitis endpoint not assessed.
Magalie Raes et al. [41]	2018	Randomized Clinical Trial	Compare minimally vs. moderately rough implants in periodontitis patients	18 patients; 84 implants; 5-year follow-up; clinical, radiographic, microbiological outcomes. Peri-implantitis incidence assessed.	Higher peri-implantitis incidence on moderately rough surfaces at 5 years. Marginal bone levels comparable but disease progression more frequent in moderately rough surfaces.
Luca Ferrantino et al. [42]	2022	Retrospective cohort study	Assess association between surface roughness, smoking and peri-implantitis	630 implants; long-term follow-up. Multilevel logistic regression analysis evaluating surface roughness, smoking, implant site location and peri-implantitis occurrence.	Rough surfaces and smoking significantly associated with higher peri-implantitis risk. Patient-related factors strongly influenced disease onset.
Badra Hussain et al. [105]	2024	Comparative Clinical Study	Compare peri-implantitis occurrence between implant systems	>600 patients; clinical and radiographic evaluation of peri-implant status; assessment of implant system characteristics.	Patient factors outweighed surface topography
Janani Gnanajothi et al. [106]	2024	Prospective Clinical Study	Evaluate inflammatory status around implants with different microgeometries	78 patients; three implant surface groups (SLA, SLActive, TiUnite). IL-1β levels in peri-implant crevicular fluid measured bu ELISA at 3 months and 1 year.	Higher IL-1β levels detected in TiUnite group compared to SLA and SLActive at both timepoints. Inflammatory markers increased over time in all groups.
Eduardo Anitua et al. [107]	2017	Controlled Retrospective Study	Assess early marginal bone stability in implants with calcium-modified surface.	Retrospective clinical and radiographic analysis of implants placed in augmented maxillary sinus.	Comparable marginal bone stability among surfaces (modified an conventional); no increased risk of early bone loss detected.
Işil Damla Şener-Yamaner et al. [108]	2017	Controlled Retrospective Study	Evaluate marginal bone loss around early-loaded SLA vs. SLActive implants.	55 patients; 157 implants. Eary loading protocols (3 vs. 8 weeks). Radiographic MBL evaluated up to long-term follow-up (>60 months).	No significant long-term differences in MBL between SLA and SLActive implants. Surface effects secondary to patient-related factors.

**Table 3:** Bias assessment.

Authors	D1	D2	D3	D4	D5	D6	D7	Overall
Luigi Cannulo et al. (2024) [98]	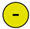	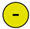	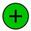	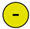	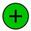	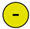	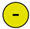	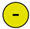
Renzo Guarnieri et al. (2019) [99]	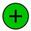	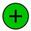	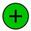	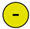	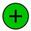	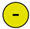	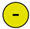	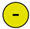
Blanca Vílchez et al. (2025) [100]	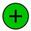	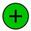	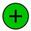	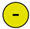	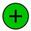	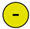	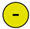	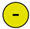
Kyung-A Ko et al. (2019) [101]	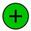	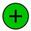	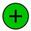	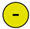	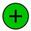	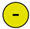	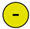	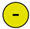
Beatrice Longhi et al. (2025) [102]	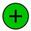	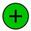	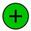	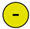	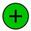	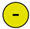	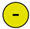	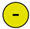
Matteo Albertini et al. (2021) [103]	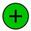	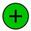	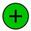	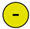	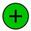	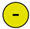	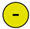	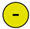
Francesco Carinci et al. (2019) [104]	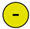	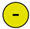	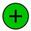	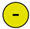	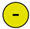	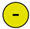	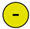	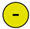
Magalie Raes et al. (2018) [41]	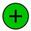	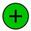	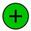	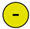	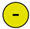	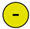	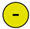	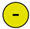
Luca Ferrantino et al. (2022) [42]	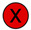	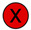	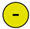	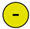	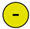	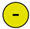	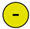	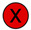
Badra Hussain et al. (2024) [105]	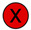	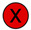	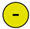	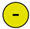	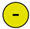	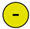	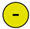	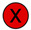
Janani Gnanajothi et al. (2024) [106]	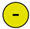	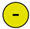	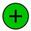	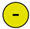	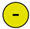	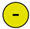	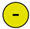	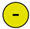
Eduardo Anitua et al. (2017) [107]	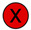	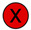	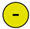	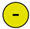	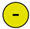	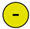	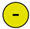	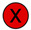
Işil Damla Şener-Yamaner et al. (2017) [108]	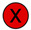	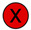	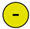	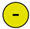	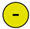	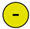	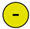	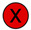
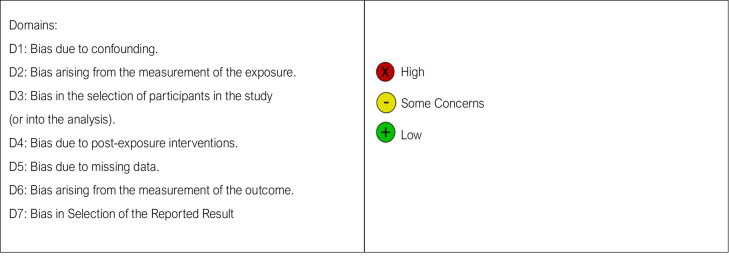								

## Data Availability

No new data were created or analyzed in this study. Data sharing is not applicable to this article.

## Abbreviations

The following abbreviations are used in this manuscript:

BIC	Bone-to-Implant Contact
ISQ	Implant Stability Quotient
PRISMA	Preferred Reporting Items for Systematic Reviews and Meta-Analyses
PROSPERO	The International Prospective Register of Systematic Reviews
RCT	Randomized Controlled Trials
SLA	Sandblasted and acid-etched
